# Tiny Language Models for Automation and Control: Overview, Potential Applications, and Future Research Directions

**DOI:** 10.3390/s25051318

**Published:** 2025-02-21

**Authors:** Ismail Lamaakal, Yassine Maleh, Khalid El Makkaoui, Ibrahim Ouahbi, Paweł Pławiak, Osama Alfarraj, May Almousa, Ahmed A. Abd El-Latif

**Affiliations:** 1 Multidisciplinary Faculty of Nador, Mohammed Premier University, Oujda 60000, Morocco; ismail.lamaakal@ieee.org (I.L.); k.elmakkaoui@ump.ac.ma (K.E.M.); i.ouahbi@ump.ac.ma (I.O.); 2 National School of Applied Sciences, Sultan Moulay Slimane University, Beni Mellal 23000, Morocco; 3 Department of Computer Science, Faculty of Computer Science and Telecommunications, Cracow University of Technology, Warszawska 24, 31-155 Krakow, Poland; pawel.plawiak@pk.edu.pl; 4 Institute of Theoretical and Applied Informatics, Polish Academy of Sciences, Bałtycka 5, 44-100 Gliwice, Poland; 5 Computer Science Department, Community College, King Saud University, Riyadh 11437, Saudi Arabia; oalfarraj@ksu.edu.sa; 6 Information Technology Department, College of Computer and Information Sciences, Princess Nourah bint Abdulrahman University, Riyadh 11586, Saudi Arabia; mmalmousa@pnu.edu.sa; 7 EIAS Data Science Lab, Center of Excellence in Quantum and Intelligent Computing, College of Computer and Information Sciences, Prince Sultan University, Riyadh 11586, Saudi Arabia; aabdellatif@psu.edu.sa; 8 Department of Mathematics and Computer Science, Faculty of Science, Menoufia University, Shebin El-Koom 32511, Egypt

**Keywords:** edge computing, IoT, model compression, optimization, small language models, tiny language models, large language models

## Abstract

Large Language Models (LLMs), like GPT and BERT, have significantly advanced Natural Language Processing (NLP), enabling high performance on complex tasks. However, their size and computational needs make LLMs unsuitable for deployment on resource-constrained devices, where efficiency, speed, and low power consumption are critical. Tiny Language Models (TLMs), also known as BabyLMs, offer compact alternatives by using advanced compression and optimization techniques to function effectively on devices such as smartphones, Internet of Things (IoT) systems, and embedded platforms. This paper provides a comprehensive survey of TLM architectures and methodologies, including key techniques such as knowledge distillation, quantization, and pruning. Additionally, it explores potential and emerging applications of TLMs in automation and control, covering areas such as edge computing, IoT, industrial automation, and healthcare. The survey discusses challenges unique to TLMs, such as trade-offs between model size and accuracy, limited generalization, and ethical considerations in deployment. Future research directions are also proposed, focusing on hybrid compression techniques, application-specific adaptations, and context-aware TLMs optimized for hardware-specific constraints. This paper aims to serve as a foundational resource for advancing TLMs capabilities across diverse real-world applications.

## 1. Introduction

### 1.1. Background

Over the past decade, Natural Language Processing has seen transformative advancements with the rise of Large Language Models [1,2]. These models, such as BERT [3], GPT-3 [4], T5 [5], and more recently, LLaMA [6] and PaLM [7], consist of hundreds of millions to billions of parameters, enabling them to perform complex linguistic tasks like text generation [8], question answering [9], summarization [10], and translation [11] with unprecedented accuracy. These LLMs are typically built on transformer architectures [12], which are trained on massive datasets and designed to capture intricate relationships within language, allowing them to understand and generate human-like text. This capacity to handle nuanced language tasks has led to widespread adoption of LLMs in various applications, from chatbots and virtual assistants to automated content creation and translation services [13].

However, the sheer size and computational requirements of LLMs present significant limitations, especially in real-time or resource-constrained applications. LLMs require substantial memory, processing power, and storage space [14], often relying on high-end GPUs or specialized hardware like TPUs (Tensor Processing Units) for both training and inference [15]. This dependency on powerful infrastructure makes LLMs primarily suited for centralized, cloud-based applications, where latency and resource costs can be managed. For example, the popular GPT-3 model [4], with 175 billion parameters, necessitates vast computing resources and is impractical to deploy directly on mobile devices, edge systems, or IoT platforms [16]. Moreover, the energy consumption of these large models is significant, contributing to environmental concerns around their large-scale use.

The need for efficient NLP solutions has spurred the development of Tiny Language Models, compact models that retain core capabilities of LLMs while reducing computational demands. TLMs are typically created by employing techniques such as knowledge distillation, pruning, quantization [17,18,19,20,21,22,23], and specialized architectures [24] designed to minimize model size and memory footprint. These methods enable TLMs to operate with a fraction of the parameters found in LLMs, making them more suitable for deployment on devices with limited resources, such as smartphones, embedded systems, and industrial IoT sensors [25].

**Figure 1:**
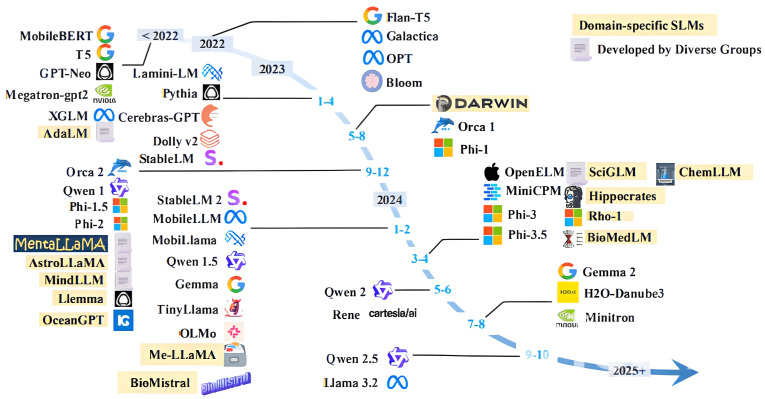
An overview of TLMs from 2022–2024 [48].

Figure 1 shows the rapid advancement of TLMs from 2022 to 2024, which has marked a transformative shift in the field of efficient NLP. Early developments in 2022 were driven by models such as MobileBERT, GPT-Neo, and T5, which laid the foundation for lightweight, resource-efficient alternatives to traditional LLMs. During 2023, significant strides were made with models like Cerebras-GPT [26], Pythia [27], Dolly v2 [28], and StableLM [29], which refined efficiency-focused architectures and optimized transformer designs to enhance performance on edge devices. Concurrently, domain-specific TLMs emerged, such as MentalLLaMA [30], AstroLLaMA [31], and OceanGPT [32], catering to specialized applications. The momentum continued in 2024, with the introduction of advanced models like Qwen 1.5 [33], TinyLlama [34], OLMo [35], and Gemma [36], which further optimized memory efficiency and inference speed. Additionally, domain-specific models such as SciGLM [37] and ChemLLM [38] demonstrated the adaptability of TLMs in scientific and biomedical fields. The increasing diversification of TLMs, including the emergence of Phi-3 [39], OpenELM [40], and MiniCPM [41], highlights the industry’s focus on delivering high-performance NLP capabilities while maintaining computational feasibility for a broad range of applications.

The significance of TLMs extends beyond just size reduction; they represent a critical step toward making advanced NLP accessible in real-time, low-latency settings where LLMs are not feasible. In domains like industrial automation [42], healthcare [43], and consumer electronics [44], TLMs allow NLP functionalities to be embedded directly within devices, enabling intelligent interactions without constant reliance on the cloud. This capability is particularly valuable in applications requiring quick response times, privacy, and offline processing, such as voice commands in smart home devices [45], diagnostics in medical devices [46], and machine control in robotics [47].

This demand for efficient language models highlights an emerging trend toward ’right-sized’ NLP models that balance performance with practical deployment requirements. While LLMs continue to push the boundaries of what is possible in NLP by handling complex and high-dimensional language tasks, TLMs are meeting a different yet equally critical need: enabling intelligent language processing within the confines of limited hardware resources. Together, these developments underscore the importance of adaptable NLP models, paving the way for broader, more versatile applications across diverse fields.

### 1.2. Paper Motivation

The motivation for this paper stems from the increasing demand for efficient NLP models that can operate on resource constrained devices such as mobile phones, IoT systems, and embedded platforms. While large language models have achieved remarkable results in NLP, their substantial computational and storage requirements make them impractical for many real-world applications where low power consumption, fast response times, and privacy are critical. TLMs offer a promising solution by balancing language processing capabilities with reduced size and resource demands, making them suitable for a broader range of applications. Despite the growing interest and advancements in TLMs, there is a noticeable lack of surveys in this specific area, leaving a gap in comprehensive resources that detail the current state, challenges, and opportunities within the field. This survey aims to address this gap by providing a thorough overview of TLMs advancements, evaluating their effectiveness in practical applications, and highlighting areas where further innovation can push the boundaries of efficient NLP on compact devices.

### 1.3. Main Contributions

This survey offers several key contributions, outlined as follows:

- **Comprehensive Survey on Tiny Language Models**: This paper presents the first comprehensive survey focused on TLMs designed specifically for NLP applications on resource-limited devices.
- **Comparison of TLM Architectures and Optimization Techniques**: This survey provides an in-depth comparison of various TLM architectures, covering model sizes, structures, and performance across applications. This includes a detailed analysis of TLM adaptations to resource constraints, such as knowledge distillation, quantization, pruning.
- **Discussion of Potential TLMs Applications in Real-World Scenarios**: This survey explores the potential applications of TLMs across a range of real-world scenarios.
- **Exploration of Challenges and Future Research Directions**: This survey addresses current limitations of TLMs, offering a discussion on emerging solutions and future research directions, including hybrid compression strategies, development of domain-specific TLMs, and context-aware adaptations for varied hardware and deployment settings.

### 1.4. Survey Structure

**Figure 2:**
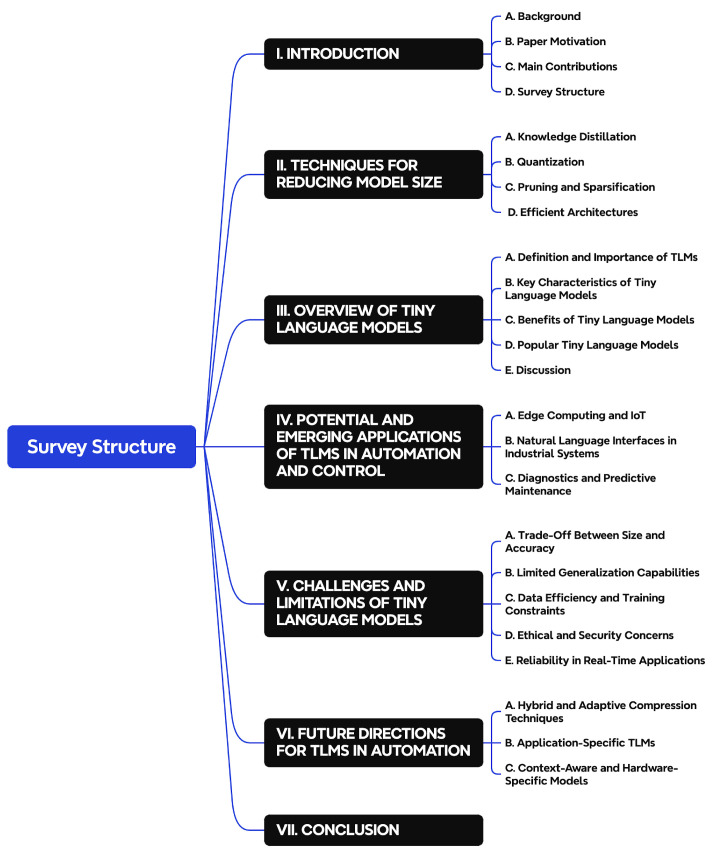
Paper Organization.

The structure of this survey is organized as follows (see Figure 2). Section 1 provides an introduction to Tiny Language Models, covering the background, motivation, and the main contributions of the paper. Section 3 offers a detailed overview of TLMs, defining their key characteristics and discussing popular TLM architectures. Section 2 describes techniques for reducing model size, including knowledge distillation, quantization, pruning, and efficient architectures. Section 4 examines the potential and emerging applications of TLMs in automation and control, focusing on edge computing, IoT, and diagnostics. Section 5 addresses the challenges and limitations of TLMs, such as the trade-off between size and accuracy. Section 6 explores future directions for TLMs in automation, including hybrid compression, application-specific models, and context-aware TLMs. Finally, Section 7 concludes the survey with closing remarks and reflections on the future potential of TLMs.

## 2. Techniques for Reducing Model Size

In this section, we discuss key techniques for reducing the size of language models, including knowledge distillation, quantization, pruning, and the design of efficient architectures. Each technique focuses on minimizing parameters while preserving model performance, making them suitable for Tiny Language Models used in resource-constrained environments.

### 2.1. Knowledge Distillation

Knowledge distillation is a technique that transfers knowledge from a larger, pre-trained *teacher model* to a smaller *student model* [49,50,51,52]. The student model is trained to mimic the teacher’s behavior, matching its predictions to achieve similar performance with fewer parameters.

The distillation loss combines task-specific loss $L_{\mathrm{task}}$ and the knowledge distillation loss $L_{\mathrm{distill}}$, which measures the KL-divergence between the teacher’s and student’s softened probability distributions. The total loss is given by:

(1) $$L=\left(1-\alpha \right)\cdot L_{\mathrm{task}}+\alpha \cdot T^{2}\cdot \mathrm{KL}\left(P_{t}\|P_{s}\right)$$

where $P_{t}=\mathrm{softmax}\left(z_{t}/T\right)$ and $P_{s}=\mathrm{softmax}\left(z_{s}/T\right)$ are the probability distributions from the teacher and student models, respectively, *T* is the temperature parameter that controls the softness of the output logits, and α is a balancing factor between the task and distillation loss.

Algorithm 1 demonstrates the process of knowledge distillation, which involves training the student model by combining the task-specific loss and the distillation loss. The algorithm begins with the teacher and student models’ forward passes to calculate logits and softened probabilities. It then computes the total loss using both components and iteratively updates the student model’s weights to minimize the combined loss. This iterative process ensures that the student model captures the teacher’s knowledge while maintaining computational efficiency.
**Algorithm 1** Knowledge Distillation1:**Input:** Teacher model $M_{t}$, Student model $M_{s}$, temperature *T*, balancing factor α2:**for** each batch of input data (x,y) **do**3:    Forward pass $M_{t}$ and $M_{s}$ to obtain logits $z_{t}$ and $z_{s}$4:    Compute softened probabilities: $P_{t}=\mathrm{softmax}\left(z_{t}/T\right)$, $P_{s}=\mathrm{softmax}\left(z_{s}/T\right)$5:    Calculate $L_{\mathrm{task}}=\mathrm{CE}\left(y,P_{s}\right)$6:    Calculate $L_{\mathrm{distill}}=T^{2}\cdot \mathrm{KL}\left(P_{t}\|P_{s}\right)$7:    Compute total loss: $L=\left(1-\alpha \right)\cdot L_{\mathrm{task}}+\alpha \cdot L_{\mathrm{distill}}$8:    Update $M_{s}$ by minimizing L9:**end for**

### 2.2. Quantization

Quantization is a technique that aims to reduce the precision of numerical representations for model parameters and activations, which, in turn, lowers memory requirements and computational demands [53,54,55,56]. The primary quantization approaches are:

- Low-Precision Floating-Point Formats:
  This approach uses lower-precision floating-point numbers instead of the typical 32-bit floating-point (FP32) representation, thus reducing memory usage while preserving a wide range of representable values. Common formats include FP16 and Bfloat16, each utilizing 16 bits [57]. A floating-point number can be expressed as:
  (2) $$x={\left(-1\right)}^{s}\times m\times 2^{e}$$
  where *s* is the sign, *m* the mantissa, and *e* the exponent. These reduced-precision formats are especially effective in model training and inference.
- Fixed-Point Representation:
  Here, numbers are stored with a fixed number of digits before and after a binary point [58], leading to faster, simpler calculations. Fixed-point representation, as opposed to floating-point, fixes the position of the binary point, making it ideal for hardware applications. In general, a fixed-point number is defined by:
  (3) $$x=\mathrm{integer}\;\mathrm{part}+\frac{\mathrm{fractional}\;\mathrm{part}}{2^{n}}$$
  where *n* denotes the bits allocated for the fractional component. Fixed-point arithmetic is highly power-efficient and works well for embedded systems.
- Binarization and Ternarization:
  These extreme quantization methods limit parameters to two or three values, respectively.
  **Binarization** [59] represents weights as either −1 or 1, computed by:
  (4) $$\hat{w}=\mathrm{sign}\left(w\right)$$
  where $\hat{w}$ is the binarized weight. This significantly reduces memory usage.
  **Ternarization** [60] maps weights to values of −1, 0, or 1, and is defined by:
  (5) $$\hat{w}=\left\{\begin{array}{cc} 1 & \mathrm{if}\;w>\Delta \\ 0 & \mathrm{if}\;|w|\leq \Delta \\ -1 & \mathrm{if}\;w<-\Delta \end{array}\right.$$
  where Δ is a set threshold.
- Logarithmic Quantization:
  This technique restricts values to powers of two, which allows for efficient storage and computation by employing simple bit-shift operations [61]. Logarithmic quantization is expressed as:
  (6) $$\hat{w}=\mathrm{round}\left(\log_{2}\left(w\right)\right)$$
  The value is scaled afterward to approximate the original parameter, providing an effective solution for energy-sensitive applications.

### 2.3. Pruning and Sparsification

Pruning reduces model complexity by removing less important weights or neurons, resulting in a sparser model with fewer parameters [62,63,64,65]. There are two main types:

- **Structured Pruning:** Removes entire channels, neurons, or layers.
- **Unstructured Pruning:** Removes individual weights based on their magnitude.

Pruning function:

(7) $$w_{p}=\left\{\begin{array}{cc} 0, & \mathrm{if}\;|w|<\tau \\ w, & \mathrm{otherwise} \end{array}\right.$$

where *w* is the weight, $w_{p}$ is the pruned weight, and τ is the pruning threshold.

Algorithm 2 describes the pruning process, where weights below a certain threshold are set to zero. This threshold-based pruning ensures that only significant weights contribute to the model’s predictions, thereby reducing computational overhead. Iterative pruning can be used to gradually achieve the desired sparsity.
**Algorithm 2** Pruning Process1:**Input:** Model weights *W*, pruning threshold τ2:**for** each layer in the model **do**3:    **for** each weight *w* in the layer **do**4:        **if** |w|<τ **then**5:           Set w=06:        **end if**7:    **end for**8:**end for**

Pruning can be applied iteratively to achieve the desired sparsity, and is particularly useful for real-time applications where speed and memory efficiency are crucial.

### 2.4. Efficient Architectures

Efficient architectures [24,66] are specially designed to minimize parameters while retaining performance. Some common architectures include:

- **TinyBERT** [67]: A reduced-layer BERT model trained via distillation.
- **MobileBERT** [68]: Incorporates bottleneck structures to reduce parameter count while maintaining capacity.

The bottleneck in MobileBERT reduces the hidden dimension size, reducing computation requirements:

(8) $$d_{\mathrm{bottleneck}}<d_{\mathrm{hidden}}$$

where $d_{\mathrm{bottleneck}}$ is the bottleneck dimension, and $d_{\mathrm{hidden}}$ is the original hidden dimension.

Algorithm 3 outlines the design principles for creating efficient architectures. It involves reducing the number of layers, introducing bottleneck structures, and optimizing attention heads and dimensions. Fine-tuning is performed to recover any performance lost during optimization.
**Algorithm 3** Efficient Architecture Design1:**Input:** Baseline model architecture2:Reduce number of layers and introduce bottleneck layers3:Optimize attention heads and dimensions for efficiency4:Fine-tune the model to recover performance

Table 1 presents a comparative analysis of key model size reduction techniques based on three major aspects: model size reduction, latency improvement, and accuracy trade-offs. The Model Size Reduction column quantifies the effectiveness of each technique in reducing storage and memory requirements, where ‘High’ corresponds to a reduction of more than 50%, ‘Moderate’ ranges between 20–50%, and ‘Minimal’ is below 20%. The Latency Improvement column represents the expected speedup in inference time, where ‘High’ indicates a speedup greater than 2×, ‘Moderate’ is between 1.2× and 2×, and ‘Minimal’ is below 1.2×. The Accuracy Trade-off column highlights the potential accuracy degradation due to applying the respective method, with ‘Minimal’ implying less than 1% accuracy loss, ‘Minor’ referring to 1–3% degradation, and ‘Variable’ indicating dependency on specific implementation details and dataset characteristics.

## 3. Overview of Tiny Language Models

This section offers a comprehensive overview of TLMs, examining their definitions, key characteristics, and popular implementations to illustrate their increasing relevance in resource-constrained environments.

### 3.1. Definition and Importance of TLMs

Tiny Language Models are streamlined natural language processing models designed to perform various linguistic tasks efficiently while utilizing significantly fewer parameters than traditional LLMs. Typically containing tens to hundreds of millions of parameters, TLMs are optimized for performance in resource-constrained environments, allowing them to function effectively on devices with limited computational power and memory.

The importance of TLMs is increasingly evident in today’s technology landscape, where there is a growing demand for intelligent applications that can operate in real-time. As industries seek to implement smart solutions across various domains, TLMs enable essential natural language processing capabilities directly on edge devices, mobile platforms, and IoT systems. This accessibility is vital for applications requiring quick response times [69], such as virtual assistants, command processing in industrial automation, and real-time data analysis in healthcare settings. By bridging the gap between sophisticated language understanding and practical deployment limitations, TLMs are becoming essential tools for enhancing user experience and operational efficiency across a wide range of applications.

### 3.2. Key Characteristics of Tiny Language Models

#### 3.2.1. Size and Complexity

TLMs are significantly smaller than LLMs, typically containing tens to hundreds of millions of parameters compared to billions in LLMs like GPT-3 [4]. This compact size allows for easier storage and faster training, enabling quicker deployment and iteration in various applications.

#### 3.2.2. Efficiency

TLMs are designed for efficiency, requiring less memory and processing power, which translates to lower energy consumption [70]. They can operate on standard CPUs or low-power GPUs, making them ideal for edge devices and resource-constrained environments. This efficiency leads to faster inference times, crucial for real-time applications such as automated command processing in industrial settings.

#### 3.2.3. Flexibility

TLMs are versatile and can be adapted for a wide range of tasks with minimal fine-tuning [71]. Their ability to function across different platforms such as mobile devices and IoT applications enhances their usability in various domains, including sentiment analysis and customer service automation.

Table 2 compares key aspects of LLMs and TLMs to highlight their differences in efficiency and application suitability.

### 3.3. Benefits of Tiny Language Models

TLMs offer significant advantages, particularly for applications constrained by limited computational resources. These benefits make TLMs increasingly appealing across a variety of deployment contexts.

*Resource Efficiency* is a core advantage of TLMs. With fewer parameters and smaller memory footprints, they require significantly less computational power and storage compared to LLMs [72]. This makes TLMs highly suitable for deployment on devices with limited hardware capabilities, such as mobile phones, embedded systems, and IoT platforms.

*Lower Latency* is another notable benefit. Due to their reduced size, TLMs can deliver faster response times, which is crucial for real-time applications in fields like healthcare, automation, and conversational AI [26,73]. Their ability to handle tasks and interactions quickly makes them ideal for scenarios where delays are not acceptable.

*Energy Savings* is an important consideration in modern applications, and TLMs excel in this aspect. By operating on less power-intensive hardware and reducing computational requirements, they help conserve energy [72]. This makes TLMs an eco-friendly option, especially beneficial for battery-operated devices or systems in remote locations where power resources are limited.

*Enhanced Privacy and Security* is a significant feature of TLMs. Since they can be deployed directly on devices, there is minimal need to send data to the cloud for processing. This on-device capability ensures that sensitive information is processed locally, reducing potential risks associated with data transmission and improving data privacy [74].

*Cost-Effectiveness* is another major advantage. The smaller infrastructure requirements of TLMs reduce the costs of running and maintaining high-performance hardware [48]. This makes advanced NLP capabilities more accessible, allowing small and medium-sized enterprises to adopt TLMs without significant financial investment.

*Scalability in Distributed Systems* is facilitated by the low-resource footprint of TLMs. They can be easily scaled across distributed systems and edge networks, enabling applications such as smart cities and industrial automation [48]. In these contexts, numerous devices operate simultaneously, relying on the efficiency of TLMs for language processing.

### 3.4. Architecture of TLMs

The architecture of TLMs is predominantly built upon the foundations established by LLMs, but it is specifically tailored for computational efficiency and scalability. At the core of most TLMs is the Transformer architecture, which is renowned for its ability to capture long-range dependencies in text through self-attention mechanisms. This makes it particularly effective for maintaining high performance while operating with constrained resources.

#### Transformer for TLMs

**Figure 3:**
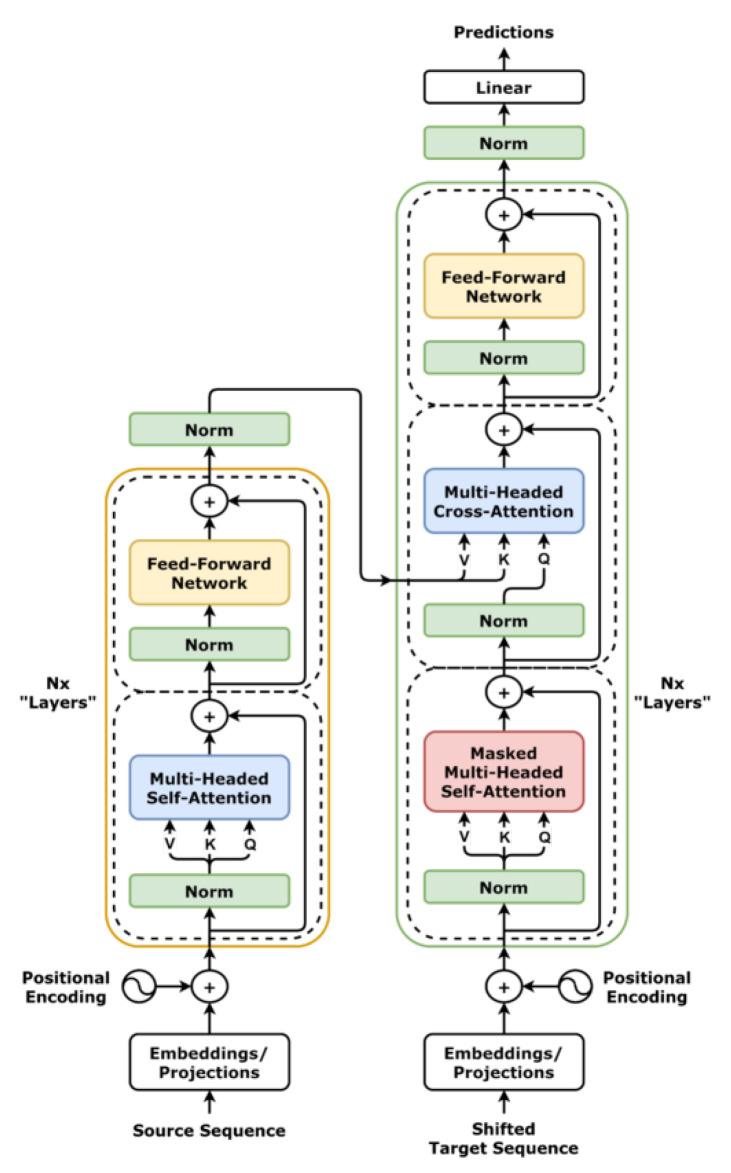
Transformer architecture.

The Transformer’s self-attention mechanism [75] enables TLMs to efficiently process sequences of varying lengths by weighing the importance of each token relative to others within the input sequence (see Figure 3). The self-attention formula is expressed as:

(9) $$\mathrm{Attention}\left(Q,K,V\right)=\mathrm{softmax}\left(\frac{QK^{T}}{\sqrt{d_{k}}}\right)V$$

where *Q*, *K*, and *V* are the query, key, and value matrices, respectively, and $d_{k}$ is the dimension of the key matrices, providing stability in the computations. The dot product $QK^{T}$ measures the similarity between the query and key, allowing the model to focus on the most relevant parts of the input.

***Multi-Head Attention (MHA) [75]*** is a key component of the Transformer model that improves its ability to focus on different parts of the sequence simultaneously. It does this by using multiple attention heads, each attending to different aspects of the sequence:

(10) $$\mathrm{MultiHead}\left(Q,K,V\right)=\mathrm{Concat}\left(head_{1},head_{2},\cdots ,head_{h}\right)W_{O}$$

Each head in the multi-head attention mechanism operates independently, allowing the model to capture different types of relationships within the sequence. The outputs from all heads are then concatenated and passed through a linear transformation.

To address the memory inefficiencies associated with traditional MHA, modifications such as ***Multi-Query Attention (MQA) [76]*** and ***Grouped Query Attention (GQA) [77]*** have been introduced. MQA shares a single set of keys and values across all attention heads, reducing memory usage. GQA takes a middle ground by grouping some query heads together and allowing them to share a set of keys and values. These optimizations reduce memory and computational costs while maintaining performance.

***Multi-Head Latent Attention (MLA) [78]*** takes this a step further by compressing the key-value pairs into a latent vector. This significantly reduces the overhead involved in managing large numbers of keys and values, while still preserving the performance of the model.

Additionally, ***Flash Attention [79,80]*** accelerates the computation of attention by minimizing memory usage, which is particularly important when working with longer input sequences. This optimization enables TLMs to process sequences more efficiently, making them more suitable for environments with tight resource constraints.

***Feedforward Networks (FFN) [81] in TLMs*** is another crucial component of Transformer-based models. It typically consists of two linear layers separated by a non-linear activation function. The formula for the feedforward network is given by:

(11) $$FFN\left(x\right)=\sigma \left(xW_{1}+b_{1}\right)W_{2}+b_{2}$$

where $W_{1}$ and $W_{2}$ are weight matrices, and $b_{1}$ and $b_{2}$ are bias terms. The activation function σ introduces non-linearity into the model, enabling it to learn complex relationships.

Commonly used activation functions in TLMs include ***ReLU (Rectified Linear Unit) [82]***, ***GELU (Gaussian Error Linear Unit) [83]***, and ***SiLU (Sigmoid Linear Unit) [84]***, each offering distinct advantages in terms of gradient flow and model expressiveness.

*ReLU* is a simple activation function widely used for its computational efficiency, defined as σ(x)=max(0,x).

*GELU* is a smoother alternative, defined as GELU(x)=x·Φ(x), where Φ(x) is the standard Gaussian cumulative distribution function (CDF). It offers better gradient flow control and is commonly used in large models like BERT and GPT.

*SiLU* combines the sigmoid function with its input, providing a smooth non-linearity that improves model performance, particularly in deeper models.

***Positional Embeddings [75]:*** Since the Transformer architecture is not inherently sequential, positional embeddings are crucial for encoding the order of tokens in a sequence. The standard approach uses sinusoidal functions to represent positions, with the following equations:

(12) $$PE\left(\mathrm{pos},2i\right)=\sin\left(\frac{\mathrm{pos}}{{10,000}^{2i/d_{\mathrm{model}}}}\right)$$

(13) $$PE\left(\mathrm{pos},2i+1\right)=\cos\left(\frac{\mathrm{pos}}{{10,000}^{2i/d_{\mathrm{model}}}}\right)$$

where pos is the token’s position in the sequence, *i* is the dimension index, and $d_{\mathrm{model}}$ is the model’s dimensionality. This technique allows the model to distinguish tokens based on their relative positions in the sequence.

***Rotary Positional Embedding (RoPE) [85]*** enhances this approach by introducing rotational transformations, improving the model’s ability to handle long-range dependencies and better understand the dynamics of token positions. Unlike traditional sinusoidal positional encodings, RoPE represents positional information by applying a rotation matrix to token embeddings, maintaining relative positional relationships. Mathematically, RoPE applies the transformation:

(14) $$\mathrm{RoPE}\left(x\right)=x\cdot e^{i\theta_{pos}}$$

where *x* is the token embedding and $\theta_{pos}$ represents the rotational angle for the given position. This transformation is applied in complex space, ensuring that relative positional differences remain consistent across layers. More explicitly, for a given position pos, the rotational embedding follows:

(15) $$\theta_{pos}=\frac{pos}{{10,000}^{\frac{2i}{d}}}$$

where *i* is the dimension index, *d* is the model’s hidden size, and pos is the position of the token. By leveraging these rotational transformations, RoPE efficiently encodes positional information, allowing transformers to generalize well across longer sequences.

***Layer Normalization and Efficiency Enhancements [86]*** stabilizes the training process by normalizing the inputs within each layer, helping to accelerate convergence. This is achieved by computing the mean and variance for each layer and normalizing the activations:

(16) $$\mathrm{LN}\left(x\right)=\frac{x-\mu }{\sigma }$$

where μ and σ are the mean and standard deviation of the input activations across the feature dimension:

(17) $$\mu =\frac{1}{N}\sum_{i=1}^{N}x_{i},\;\sigma =\sqrt{\frac{1}{N}\sum_{i=1}^{N}{\left(x_{i}-\mu \right)}^{2}+\epsilon }$$

To enhance model flexibility, ***Parametric Layer Normalization (PLN) [87]*** introduces learnable parameters γ and β for adaptive scaling and shifting:

(18) $$\mathrm{PLN}\left(x\right)=\gamma \cdot \frac{x-\mu }{\sigma }+\beta$$

Additionally, ***RMS Normalization (RMSNorm) [88]*** simplifies the calculation by using the root mean square of inputs instead of standard normalization, which reduces computational demands:

(19) $$\mathrm{RMSNorm}\left(x\right)=\gamma \cdot \frac{x}{\sqrt{\frac{1}{N}\sum_{i=1}^{N}x_{i}^{2}+\epsilon }}+\beta$$

where ϵ is a small constant added for numerical stability. These normalization techniques significantly improve training stability, reduce vanishing gradients, and enhance computational efficiency, particularly in small language models.

### 3.5. Common Training Datasets for TLMs

This section explores the open-sourced pre-training datasets commonly utilized in training TLMs. Our analysis identifies 12 datasets employed in these efforts:

1. RefinedWeb [89]: A high-quality dataset sourced from CommonCrawl, carefully filtered to ensure the retention of valuable web content.
2. CulturaX [90]: A comprehensive multilingual dataset spanning 167 languages, designed for cross-cultural and multilingual model training.
3. FineWeb-Edu [91]: An educationally focused dataset derived from the broader FineWeb corpus, specifically curated for instructional content.
4. The Pile [92]: A diverse mixture of smaller datasets encompassing multiple domains, making it a foundational resource for pretraining.
5. RedPajama [93]: A dataset comprising over 100 billion text documents, extracted from 84 CommonCrawl snapshots and processed via the CCNet pipeline.
6. Cosmopedia [94]: A synthetic dataset featuring textbooks, stories, blogs, WikiHow articles, and posts, generated using the Mixtral-8x7B-Instruct-v0.1 model.
7. RoBERTa [95] CCNewsV2: A dataset that includes an updated version of the English text from the CommonCrawl News corpus, specifically curated for training robust models.
8. WuDaoCorpora [96]: A massive Chinese corpus with approximately 3 trillion tokens and 1.08 trillion Chinese characters, designed for large-scale language modeling.
9. DCLM-baseline [97]: Built from Common Crawl data, this dataset includes effective pretraining strategies using the OpenLM framework, along with evaluations across 53 downstream tasks.
10. Dolma [98]: An English-language corpus that employs MinHash algorithms for deduplication both within and across datasets.
11. StarCoder [99]: A domain-specific dataset focused on Python programming language tokens, suitable for code-related language modeling tasks.
12. PushShift.io Reddit [100]: A social media archive containing Reddit data collected since 2015, aimed at enabling social media analysis and research.

These datasets collectively represent a diverse array of linguistic resources, ranging from general-purpose web corpora to specialized domain-specific datasets, enabling efficient and comprehensive pretraining of TLMs.

### 3.6. Popular Tiny Language Models

TLMs have gained traction due to their ability to deliver efficient and effective natural language processing in resource-constrained environments. This subsection highlights some of the most prominent TLMs in the literature, outlining their unique features, architectures, and applications in various domains.

#### 3.6.1. Transformer-Based Encoder-Only Models

These models are built using the transformer architecture with an encoder-only design, making them suitable for tasks like text classification, sentiment analysis, and named entity recognition.

***DistilBERT***: A distilled version of BERT designed to be smaller and faster while retaining much of its performance, containing approximately 66 million parameters with six transformer layers instead of twelve [101]. Its architecture is optimized for efficiency, making it ideal for applications such as sentiment analysis, text classification, and named entity recognition, particularly in environments with limited computational resources.

***TinyBERT***: Employs a two-stage training method combining knowledge distillation and task-specific fine-tuning to create a compact model with around 14.5 million parameters [67]. This architecture retains essential language understanding capabilities while being lightweight, making TinyBERT suitable for various NLP tasks such as question answering and sentiment classification, especially in mobile and embedded systems.

***MobileBERT***: Is optimized for mobile devices, achieving BERT-like performance with approximately 25.3 million parameters while being about 50% smaller and faster due to its bottleneck architecture. It is task-agnostic, allowing for easy fine-tuning across various NLP tasks. MobileBERT is 4.3× smaller and 5.5× faster than BERTBASE, with a GLUE score of 77.7 and a latency of 62 ms on a Pixel 4 phone. It also achieves competitive F1 scores on the SQuAD v1.1/v2.0 tasks, making it suitable for efficient NLP applications on resource-limited mobile platforms [68].

***MiniLM***: A compact transformer model with approximately 33 million parameters that utilizes self-attention mechanisms for efficient contextual understanding [102]. Its architecture is designed for both speed and performance, making it effective for various NLP tasks, including semantic similarity and question answering, particularly in scenarios requiring rapid processing on limited hardware.

***SqueezeBERT***: An efficient NLP model designed for mobile and resource-constrained devices, aiming to retain the performance of large models like BERT while significantly reducing computational requirements. By replacing certain operations in BERT’s self-attention layers with grouped convolutions, an approach commonly used to speed up computer vision models, SqueezeBERT achieves a 4.3× speedup over BERT-base on devices like the Pixel 3, while maintaining competitive accuracy on benchmark tasks such as the GLUE test set [103]. This makes SqueezeBERT an ideal choice for on-device NLP applications like text classification, enhancing user experiences on smartphones and other mobile platforms.

***SmolLM***: A family of small, high-performance language models, available in sizes of 135 million, 360 million, and 1.7 billion parameters, designed to operate efficiently on local devices [104]. These models are built using a meticulously curated training dataset called SmolLM-Corpus, which combines synthetic textbooks and stories (Cosmopedia v2), educational Python samples, and deduplicated educational web content from FineWeb. SmolLM’s architecture leverages advanced techniques in data curation and training to maximize efficiency and accuracy, making it ideal for applications like text completion, language translation, and educational tools, particularly on devices with limited resources. SmolLM models outperform other models in their size range across benchmarks in common sense reasoning and general knowledge, supporting a range of real-world tasks with high efficiency and privacy on local devices.

#### 3.6.2. Transformer-Based Decoder-Only Models

These models use a decoder-only transformer architecture, making them ideal for text generation, conversational AI, and other generative tasks.

***TinyLLama:*** A 1.1 B parameter model that focuses on adapting large language model architectures for resource-constrained environments, maintaining usability in conversational and interactive applications. With a design optimized for smaller sizes, it retains strong performance in generating coherent responses, making it highly relevant for applications in chatbots and interactive systems where traditional large models would be impractical [34].

***Llama 3***: Developed by Meta, features 8 billion parameters and is engineered to deliver BERT-like performance with enhanced efficiency and speed. This model is built on a transformer architecture that has been optimized for both training and inference, making it suitable for real-time applications [105]. Llama 3 is versatile, supporting various NLP tasks such as text generation, summarization, and conversational agents. Its compact design allows for deployment in diverse environments, including mobile and web applications.

***Phi-3***: Created by Microsoft, it is notable for its flexible architecture, with parameter counts ranging from 3.8 billion to 7 billion [39]. This adaptability allows developers to tailor the model to specific application needs, optimizing performance based on resource availability. The architecture of Phi-3 builds on the transformer framework, ensuring efficient processing and effective natural language understanding. It excels in applications such as sentiment analysis, question answering, and dialogue systems.

***Gemma***: Developed by Google, Gemma operates with parameters ranging from 2 billion to 7 billion, focusing on scalability and effectiveness for a variety of NLP tasks. Utilizing a transformer architecture optimized for speed, Gemma maintains competitive performance while remaining manageable in size [36]. Its design allows for easy adaptation to various applications, including machine translation, summarization, and chatbots. Gemma is particularly suited for environments with limited computational resources, such as mobile devices and IoT systems, where efficient language processing is essential.

***Mixtral 8x7B***: Created by Mistral AI, features 7 billion parameters and is designed to enhance performance while minimizing the resource footprint of language models. This model employs a transformer-based framework with optimizations that prioritize inference speed and reduced latency, making it ideal for real-time applications [73]. Mixtral 8x7B is effective in tasks requiring quick processing, such as real-time translation, conversational AI, and content generation. Its efficiency and performance make it suitable for deployment in enterprise environments where responsiveness is critical.

***OpenELM***: Developed by Apple, is a smaller model with a parameter count ranging from 0.27 billion to 3 billion, focusing on delivering efficient performance for resource-constrained environments [40]. Its architecture simplifies traditional models to prioritize ease of deployment and low latency while retaining essential NLP capabilities. OpenELM is particularly useful for applications in mobile devices, such as virtual assistants and on-device language processing. Its lightweight design ensures fast, real-time responses, making it an excellent choice for everyday applications where efficiency is paramount.

***MobiLlama***: Designed with 0.5 billion parameters, focusing on efficiency for resource-constrained devices while challenging the “bigger is better” paradigm in natural language processing. Its architecture incorporates a careful parameter-sharing scheme derived from a larger model, which effectively reduces pre-training and deployment costs without sacrificing accuracy [106]. MobiLlama is particularly suited for applications requiring on-device processing, such as virtual assistants, chatbots, and mobile applications, where energy efficiency, low memory usage, and quick response times are essential for maintaining privacy and enhancing user experience.

***Stable LM 2 1.6 B***: This model, developed by Stability AI, is a 1.6 billion-parameter language model designed as a decoder-only transformer, inspired by the LLaMA architecture with modifications for improved efficiency [107]. Trained on 2 trillion tokens from a diverse set of open-source multilingual and code datasets over two epochs, it incorporates enhancements like Rotary Position Embeddings and an optimized tokenizer (Arcade100k) to improve throughput and tokenization of digits. This model serves as a foundational base for downstream applications and can be fine-tuned for specific tasks, making it suitable for text generation, interactive AI, and code assistance applications. Intended for general-purpose NLP, Stable LM 2 1.6 B requires fine-tuning for safe use, as it may exhibit biases or unsafe behavior if directly deployed without customization.

***Orca 2***: A 13-billion-parameter small language model focused on enhancing reasoning capabilities by training with diverse solution strategies rather than strictly imitating larger models. Building on the foundation of Orca 1, which learned from rich signals like explanation traces, Orca 2 [108] incorporates various reasoning techniques—such as step-by-step reasoning, recall then generate, and direct answering—to adapt its approach depending on the task. This flexibility enables Orca 2 to excel in complex, zero-shot reasoning tasks without solely mimicking the outputs of more capable models. Evaluated across 15 benchmarks with around 100 tasks, Orca 2 demonstrates performance comparable to much larger models, proving effective in tasks that demand advanced reasoning.

***Architext GPT-J-162M***: A 162-million-parameter transformer model specifically fine-tuned for generating architectural layouts based on natural language prompts. Built with 12 layers, a model dimension of 768, and 16 attention heads, Architext uses Rotary Position Embedding (RoPE) to handle geometric and spatial prompts effectively [109]. Initially pre-trained on the large-scale Pile dataset, the model was further fine-tuned on a procedurally generated dataset of architectural layouts created with Rhinoceros/Grasshopper, making it adept at producing diverse and structured residential floor plans. While designed to support architectural design workflows, it is best suited for generating conceptual layouts that illustrate room arrangements, adjacency, and orientation in response to descriptive prompts. However, its outputs are conceptual and not intended for precise construction documentation. Architext enables architects and designers to explore spatial configurations quickly, enhancing the early design process.

***SantaCoder***: A 1.1B-parameter code language model developed as part of the BigCode project, aimed at generating code for programming languages such as Python, JavaScript, and Java [110]. SantaCoder is optimized for both left-to-right generation and infilling tasks, showcasing strong performance on the MultiPL-E benchmark for code generation.

***TeenyTinyLlama (TTL)***: A small, open-source language model specifically tailored for Brazilian Portuguese. TTL is developed with a compact architecture, having less than 2 billion parameters, making it suitable for low-resource environments. It consists of two models with parameter sizes of 160 M and 460 M, leveraging a custom pre-training dataset named Pt-Corpus-Instruct, which combines open-source Brazilian Portuguese text with instruction-following data [111]. This design enables TTL to handle language generation and instruction-based tasks, offering a resource-efficient solution for NLP applications in underrepresented languages, particularly for local or constrained computing environments.

***Chinese Tiny LLM (CT-LLM)***: A model with 2 billion parameters designed specifically for Chinese language understanding and multilingual adaptability. CT-LLM was trained on a vast dataset of 1200 billion tokens, comprising 800 billion Chinese tokens, 300 billion English tokens, and 100 billion code tokens, emphasizing Chinese language proficiency [112]. The model architecture follows a transformer decoder structure, optimized with Rotary Position Embeddings and RMSNorm. It employs supervised fine-tuning (SFT) and preference alignment techniques to enhance performance in both Chinese and English. The model demonstrates strong results in various benchmarks, including CHC-Bench, a multidisciplinary benchmark that evaluates instruction-following in complex Chinese language tasks.

#### 3.6.3. Transformer-Based Encoder-Decoder Models

These models combine both encoder and decoder components, enabling them to handle a wide range of tasks, including translation, summarization, and question answering.

***T5-Small***: A compact version of the T5 (Text-To-Text Transfer Transformer) model developed by Google Research, designed to unify various NLP tasks within a text-to-text framework [5]. Despite its reduced parameter count, T5-Small retains the effectiveness of the larger T5 models through an encoder-decoder Transformer architecture and pre-training on a large, diverse dataset (C4). This design allows T5-Small to handle tasks like summarization, translation, and classification efficiently, making it suitable for applications on resource-constrained devices that benefit from a versatile, low-compute language model.

***CodeT5+***: An adaptable encoder-decoder language model family designed for code-related tasks, addressing the limitations of existing code LLMs that rely solely on encoder only or decoder-only architectures [113]. Unlike traditional models, CodeT5+ enables flexible module combinations tailored for different tasks, enhancing its versatility across applications. The model is pre-trained with a diverse set of objectives including span denoising, contrastive learning, text-code matching, and causal language modeling on both unimodal and bimodal multilingual code datasets, which mitigates performance issues in task-specific scenarios. By initializing with pre-trained, frozen LLMs, CodeT5+ scales efficiently, and instruction-tuning aligns the models with natural language prompts. Evaluated on over 20 benchmarks, CodeT5+ achieves state-of-the-art performance in code generation, completion, mathematical programming, and text-to-code retrieval, with the instruction-tuned 16 B model establishing new records on the HumanEval benchmark for code generation.

#### 3.6.4. Multimodal Models

These models are designed to process both textual and visual data, making them suitable for vision-language tasks such as visual question answering and image captioning.

***MobileVLM***: A mobile-oriented multimodal vision language model (MMVLM) designed to efficiently process both text and visual data. It incorporates two versions with 1.4 billion and 2.7 billion parameters, utilizing a CLIP-like pre-training approach to enhance cross-modality interactions through an efficient projector. Evaluations on standard benchmarks show that MobileVLM performs comparably to larger models while achieving impressive inference speeds of 21.5 tokens per second on Qualcomm Snapdragon 888 CPUs and 65.3 tokens per second on NVIDIA Jetson Orin GPUs [114].

***TinyGPT-V***: An accessible, open-source multimodal language model (MLLM) designed for efficient vision-language tasks, such as visual question answering, image captioning, and object recognition, while keeping computational demands low. Integrating the 2.8 billion-parameter Phi-2 language model with pre-trained vision encoders, it uses a specialized mapping module to effectively merge visual and linguistic data [115]. Tailored for smaller computational backbones, TinyGPT-V operates with only 24 GB of memory for training and 8 GB for inference, making it practical for devices with limited resources. Applications of TinyGPT-V extend to real-time analysis in augmented reality, mobile-based visual assistance for visually impaired users, and on-device content moderation for social media. Through advanced quantization techniques, TinyGPT-V matches the performance of larger models on vision-language benchmarks, offering a balanced solution between efficiency and effectiveness in real-world scenarios.

#### 3.6.5. Specialized Architectures

These models employ specialized architectures or optimizations tailored for specific tasks or environments.

***Cerebras-GPT***: is a family of open compute-optimal language models that range from 111 million to 13 billion parameters, designed to utilize recent advancements in efficient pre-training and scaling techniques [26]. These models are trained on the Eleuther Pile dataset following DeepMind’s Chinchilla scaling rules, ensuring high accuracy relative to their compute budget and benefiting from predictable power-law scaling. The architecture also incorporates Maximal Update Parameterization, which enhances model performance by improving accuracy and hyperparameter predictability at scale. Cerebras-GPT demonstrates state-of-the-art training efficiency for both pre-training and downstream applications, making it suitable for tasks like text generation and conversational AI.

***Pythia***: Developed by EleutherAI, Pythia is a suite of transformer-based language models ranging from 70 million to 12 billion parameters, tailored for interpretability research on large language models. Each model size has two versions: one trained on the Pile dataset, and one on a deduplicated version, allowing for controlled studies on model behavior and scaling effects [27]. With consistent architecture and hyperparameters across model sizes, Pythia supports systematic experiments, and its extensive checkpoints (154 per model) enable in-depth analysis at various training stages. Though primarily intended for research, Pythia can be fine-tuned for specific tasks, but it is not optimized for interactive applications like chatbots.

***DistilGPT-2***: Developed by Hugging Face, this is a distilled version of GPT-2 with approximately 66 million parameters, making it significantly smaller while retaining much of GPT-2’s functionality. Trained through knowledge distillation, DistilGPT-2 [116] operates as a “student” model that learns to mimic the behavior of the larger GPT-2 “teacher” model. This model is well-suited for tasks like interactive chatbots, automated responses, and text completion, particularly in scenarios where low latency and reduced memory usage are critical.

***GPT-Neo 125 M***: A transformer-based language model developed by EleutherAI, designed as a smaller-scale replication of GPT-3 with 125 million parameters. Trained on the extensive Pile dataset for 300 billion tokens over 572,300 steps, GPT-Neo 125 M uses a masked autoregressive language modeling approach with cross-entropy loss [117]. This model is particularly adept at generating coherent text from prompts, leveraging an internal representation of the English language to perform well in text generation and related NLP tasks. While it can be fine-tuned for various downstream tasks, it is most effective in scenarios involving text completion and prompt-based generation.

***Qwen2***: The latest addition to the Qwen series of language and multimodal models, designed with scalability and diverse deployment needs in mind. Built on the Transformer architecture and trained using next-token prediction, Qwen2 includes both foundational language models and instruction-tuned variants, enabling it to perform well in conversational, instructional, and agent-based tasks. The Qwen2 family includes models with parameter counts of 0.5 billion, 1.5 billion, 7 billion, and 72 billion, as well as a Mixture-of-Experts (MoE) model with 57 billion parameters, 14 billion of which activate per token. The smaller models, Qwen2–0.5 B and Qwen2–1.5 B, are optimized for portable devices such as smartphones and smart wearables, making them ideal for on-device NLP applications [118]. Pre-trained on a massive dataset of 7 trillion tokens covering diverse domains, Qwen2 offers enhanced code and math reasoning abilities compared to its predecessors. Through supervised fine-tuning and direct preference optimization, Qwen2 aligns well with human preferences, performing strongly on benchmarks in both general language tasks and instruction-following capabilities.

***GPT-4o Mini***: A small, cost-efficient language model with a 128K token context window, supporting up to 16K output tokens per request [119]. It achieved 82% on the MMLU benchmark, surpassing GPT-3.5 Turbo in chat preferences, and is optimized for low-latency, multi-call applications. Priced at 15 cents per million input tokens and 60 cents per million output tokens, it supports text and vision inputs, with future expansions planned for audio, video, and image outputs. Built-in safety features include RLHF and an instruction hierarchy to resist prompt injections, making it reliable for scaled applications.

Table 3 presents a detailed comparison of popular TLMs in the literature.

### 3.7. Discussion

The evolution of TLMs reflects a growing emphasis on *efficiency*, *accessibility*, and *adaptability* in NLP.

#### 3.7.1. Optimization Strategies and Architectural Innovations

A key development in TLMs is the shift toward **specialized architectures** that optimize model efficiency while maintaining performance. Unlike traditional LLMs, which rely on large parameter counts and high-dimensional embeddings, TLMs employ parameter-efficient architectures, reduced-depth transformer layers, and optimized attention mechanisms to achieve competitive accuracy with significantly fewer resources.

**Model pruning and quantization**, for instance, are widely used to compress models while preserving performance. Techniques like structured pruning eliminate redundant neurons or attention heads, while unstructured pruning removes individual weights based on their importance scores. Quantization techniques, such as *Post-Training Quantization* and *Quantization-Aware Training*, reduce the numerical precision of model parameters from FP32 to INT8 or lower, significantly lowering memory and computational overhead.

Moreover, models such as MobileBERT and SqueezeBERT employ bottleneck layers and grouped convolutions, leading to a 3× to 4× reduction in latency while maintaining >90% of the original model’s accuracy. Efficient attention mechanisms like *Grouped-Query Attention* and *Multi-Query Attention* further reduce inference costs by sharing key-value pairs across multiple attention heads, lowering memory usage while sustaining model effectiveness.

#### 3.7.2. Deployment and Adaptability Across Diverse Environments

One noteworthy trend in TLM development is the **customization of architectures for deployment environments**. Unlike generic LLMs, which require large-scale cloud infrastructure, many TLMs are specifically optimized for edge computing, mobile devices, and real-time applications.

For example:

- **MobileBERT** achieves a 4× speedup over BERT on mobile CPUs by incorporating bottleneck transformers and low-rank matrix decomposition.
- **TeenyTinyLlama** demonstrates the potential of ultra-compact models for on-device AI assistants, reducing latency by over 60% compared to standard lightweight models.
- **MobileVLM**, a multimodal variant of TLMs, extends small-scale transformer models to handle vision-language tasks, making it particularly useful for augmented reality (AR) and real-time visual assistance.

**Table 3:** Comparison of TLM Architectures (Parts 1 and 2).

					Part 1					
Model Name	Date	Hidden Size	Size	Layer Number	Head Num	Attention	Activation	Vocab. Size	Max Context Window	Open Training Datasets
Pythia [27]	2023	768	160 M	12	12	MHA	GELU	50k	2k	✔
		1024	410 M	24	16					
		2048	1 B	16	8					
		2048	1.4 B	24	16					
		2560	2.8 B	32	32					
Bloomz [120]	2022	1536	1.1 B			MHA				
		1024	560 M		16		GELU, tanh	251k	2k	✔
Bloom [121]	2022	1024	560 M	24	16	MHA	GELU, tanh	251k	2k	✔
		1536	1.1 B	24						
OPT [122]	2022	768	125 M	12	12	MHA	ReLU	50k	2k	✔
		1024	350 M	24	16					
		2048	1.3 B	24	32					
		2560	2.7 B	32	32					
Cerebras-GPT [114]	2023	768	111 M	10	12	MHA	GELU	50k	2k	✔
		1088	256 M	14	17					
		1536	590 M	18	12					
		2048	1.3 B	24	16					
		2560	2.7 B	32	32					
Galactica [123]	2022	768	125 M	12	12	MHA	GELU	50k	2k	
		2048	1.3 B	24	32					
Phi-3.5-mini	2024	3072	2.7 B	32	32	MHA	SiLU	32k	4k	
Phi-3-mini [124]	2024	3072	3.8 B	32	32	MHA	SiLU	32k	4k	
Phi-2 [125]	2023	2560	2.7 B	32	32	MHA	GELU, tanh	51k	2k	
Phi-1.5 [126]	2023	2048	1.3 B	24	32	MHA	GELU, tanh	51k		
Phi-1 [127]	2023	2048	1.3 B	24	32	MHA	GELU, tanh	51k	2k	
StableLM-2-zephyr [29]	2024	2048	1.6 B	24	32	MHA	SiLU	100k	4k	✔
StableLM-zephyr [128]	2023	2560	3 B	32	32	MHA	SiLU	50k	1k	✔
MobilLlaMA [129]	2023	2048	1.4 B	24	16	GQA	SiLU	32k	2k	✔
TinyLlama [34]	2023	2048	1.1 B	22	32	GQA	SiLU	32k	2k	✔
MobiLlama [106]	2024	2048	0.5 B	22	32	GQA	SiLU	32k	2k	✔
			1 B							
Gemma [36]	2024	2048	2 B	18	8	MQA	GELU	256k	8k	
recurrentGemma [130]	2024	2560	2 B	26	10	MQA	GELU, tanh	256k	8k	
Gemma-2 [131]	2024	2304	2 B	26	8	GQA	GELU, tanh	256k	8k	
LaMini-GPT [132]	2023	1280	774 M	36	20	MHA	GELU, tanh	50k	1k	
		1600	1.5 B	48	25					
MiniCPM3 [133]	2024	2560	4 B	62	40	MLA	SiLU	73k		
MiniCPM [41]	2024	1536	1 B	52	24	GQA	SiLU	73k	128k	
		2304	2 B	40	36			123k	131k	
**Model Name**	**Date**	**Layer Number**	**Hidden Size**	**Size**	**Head Num**	**Attention**	**Activation**	**Vocab. Size**	**Max Context Window**	**Open Training Datasets**
Toyota DCLM [134]	2024	24	2048	1.4 B	16	MHA	SiLU	50k	50k	✔
SmolLM [104]	2024	30	576	135 M	9	GQA	SiLU	49k	2k	✔
		32	960	360 M	15	GQA		49k	2k	✔
		24	2048	1.7 B	32	MHA				
AllenAI OLMo [35]	2024	16	2048	1.18 B	16	MHA	SiLU	50k	50k	✔
OpenELM [40]	2024	16	1280	270 M	12–20	GQA	SiLU	32k	2k	✔
		20	1536	450 M	12–24			32k	2k	✔
		28	2048	1.1 B	16–32			32k	2k	✔
		36	3072	3 B	12–24			32k	2k	✔
DataBricks Dolly-v2 [28]	2023	32	2560	3 B	32	MHA	GELU	50k	2k	
Danube3 [135]	2024	16	1536	0.5 B	16	GQA	SiLU	32k	8k	
		24	3840	4 B	32		SiLU	32k	8k	
Qwen 2.5 [136]	2024	24	896	0.5 B	14	GQA	SiLU	152k	32k	
		28	1536	1.5 B	12			152k	32k	
		36	2048	3 B	16			152k	32k	
Qwen 2 [137]	2024	24	2048	1.8 B	16	MHA	SiLU	152k	32k	
		40	2560	4 B	20			32k		
Qwen 1.5 [33]	2024	24	1024	0.5 B	16	MHA	SiLU	152k	32k	
Qwen 1 [138]	2023	24	2048	1.8 B	16	MHA	SiLU	152k	8k	
Fox [139]	2024	32	2048	1.6 B	16	GQA	SiLU	32k	8k	

#### 3.7.3. TLMs for Linguistic and Regional Accessibility

Another crucial advancement is the role of TLMs in expanding language accessibility across diverse linguistic and regional contexts. While most LLMs are trained predominantly on English corpora, TLMs are increasingly being fine-tuned for low-resource languages, ensuring broader adoption.

- **Chinese Tiny LLM** leverages multi-stage distillation to retain Chinese linguistic richness while reducing parameter count by 80%, making it viable for government and business applications.
- **IndicTLM**, a small-scale NLP model trained on Indian languages, employs subword tokenization and corpus filtering to optimize performance across more than 10 Indian languages, achieving state-of-the-art results on low-resource benchmarks.

#### 3.7.4. TLMs in Domain-Specific Applications

TLMs have demonstrated exceptional flexibility in **domain-specific applications**, where large-scale generic models often fail to generalize effectively. Several specialized TLMs have been optimized for targeted use cases:

- **Architext GPT-J-162M**: Trained on architectural datasets, enabling *automated floor plan generation* based on textual inputs.
- **CodeT5+**: A lightweight model designed for code generation, debugging, and auto-completion, with quantized variants achieving a 2.5× acceleration in inference speed.
- **SciTinyBERT**: Fine-tuned on scientific literature, optimized for research paper summarization and question answering.

These examples showcase the practical benefits of TLMs beyond just size reduction. Their efficiency allows them to be deployed on specialized hardware, such as microcontrollers, edge TPU accelerators, and mobile processors, where conventional LLMs remain impractical.

## 4. Potential and Emerging Applications of TLMs in Automation and Control

This section discusses key application areas where TLMs are transforming automation and control.

### 4.1. Edge Computing and IoT

TLMs are widely integrated into edge computing [140] and IoT environments [141], allowing devices to process and interpret natural language commands locally, without reliance on cloud infrastructure. This reduces latency, enhances data privacy, and minimizes power consumption.

**Smart Home Assistants:** TLMs enable on-device natural language understanding, allowing smart home devices to interpret voice commands, automate routines, and interact with users efficiently [142]. These models can be embedded in devices such as thermostats, security systems, smart lighting, and voice assistants, enabling them to function even in offline or low-connectivity conditions.

**Wearable and Health Monitoring Devices:** In smartwatches and fitness trackers, TLMs support real-time natural language interactions, allowing users to query health metrics such as heart rate, activity levels, or sleep quality [143,144]. Additionally, TLMs enable personalized health insights, generating natural language summaries of medical data and assisting in early disease detection through on-device AI processing.

**Industrial IoT Monitoring:** In factories, manufacturing plants, and industrial automation, TLMs process sensor data [145], status messages, and operational logs to provide real-time insights and alerts. These models allow IoT gateways to detect anomalies, predict failures, and generate natural language summaries, improving decision-making in industrial workflows.

**Agriculture and Environmental Monitoring:** TLMs are deployed in smart agriculture systems to interpret sensor data on soil moisture [146], temperature, and humidity, allowing automated irrigation systems to respond to natural language commands. In environmental monitoring, TLMs enable IoT sensors to analyze and summarize air quality, climate trends [147], or pollution levels, making real-time recommendations based on processed data.

### 4.2. Natural Language Interfaces in Industrial Systems

TLMs enhance human–machine interaction in industrial environments, allowing operators to interact with machines, sensors, and control systems through spoken or written commands. This improves efficiency, accessibility, and ease of use in complex automation settings.

**Voice-Controlled Machine Operations:** TLMs enable workers to operate machinery using natural language commands, reducing the need for manual input [148]. For example, industrial robots, conveyor belts, and assembly-line machines can be controlled via spoken instructions, improving accessibility and reducing operational complexity.

**Automated Industrial Reporting:** Industrial systems generate vast amounts of sensor logs, performance data, and maintenance records. TLMs are capable of processing this structured data and converting it into readable, natural language summaries for engineers and operators, improving decision-making and reducing manual reporting efforts [149].

**Multilingual Command Interpretation:** In global manufacturing plants, workers may communicate in multiple languages. TLMs facilitate real-time language translation and multilingual command processing, allowing seamless interaction with industrial systems across diverse linguistic backgrounds [13].

**Virtual Assistants for Training and Support:** TLMs serve as interactive training assistants, helping new employees learn how to operate equipment or troubleshoot issues by providing step-by-step instructions [40]. Workers can ask technical questions, and the model can generate detailed, real-time explanations to assist in problem resolution.

### 4.3. Diagnostics and Predictive Maintenance

TLMs are increasingly used in predictive maintenance and diagnostics, where they analyze machine-generated logs, system errors, and operational data to detect potential failures before they occur.

**Automated Log Analysis:** Industrial machines produce extensive logs that contain diagnostic and operational data. TLMs can analyze and summarize these logs [145], highlighting critical warnings, anomalies, or performance trends that require attention. This allows engineers to identify potential failures faster and optimize maintenance schedules.

**Fault Detection and Prediction:** By analyzing historical sensor data and real-time system outputs, TLMs can detect early signs of equipment malfunction and predict when components may fail [150]. This proactive approach helps industries prevent unexpected breakdowns and schedule maintenance efficiently.

**Interactive Maintenance Assistance:** TLMs are integrated into smart maintenance systems to provide real-time troubleshooting guidance. Technicians can input error codes or system alerts, and the model can interpret the issue and suggest corrective actions in natural language [150].

**Error Code Interpretation:** Many industrial and automotive systems generate complex diagnostic codes that require manual interpretation [113]. TLMs can process these error codes and provide plain-language explanations, helping technicians understand and address issues more effectively.

Table 4 summarizes the various domains where TLMs are making an impact in automation and control, highlighting their ability to enhance efficiency, enable real-time interaction, and improve decision-making in industrial and IoT environments.

## 5. Challenges and Limitations of Tiny Language Models

While Tiny Language Models offer significant advantages in terms of efficiency and deployment on resource-constrained devices, they also present a unique set of challenges and limitations.

### 5.1. Trade-Off Between Size and Accuracy

Reducing the size of language models often results in a compromise on accuracy and performance, as TLMs with fewer parameters may not capture the same level of linguistic nuance or contextual depth as larger models like GPT-4 or BERT-large. This limitation is particularly evident in complex language tasks, such as intricate question answering [151] or nuanced sentiment analysis [152]. To address this, hybrid compression techniques, which combine knowledge distillation with task-specific fine-tuning [153], can be implemented to improve TLMs’ performance on complex tasks. Additionally, selective parameter expansion for key layers may enhance accuracy without significantly increasing model size [154]. Using transfer learning, where smaller models are pre-trained on large-scale datasets and later fine-tuned on specific tasks [155], can help bridge the accuracy gap. Another approach is the integration of attention-based mechanisms [156] that selectively allocate more computational resources to challenging segments of the input text, thereby preserving model performance without expanding the entire model’s size.

### 5.2. Limited Generalization Capabilities

Due to their reduced size, TLMs may show limited generalization across diverse tasks or domains without extensive fine-tuning, restricting their usability in applications requiring versatile language understanding and adaptability. Domain-adaptive pretraining [157] can help TLMs generalize more effectively across various tasks, while meta-learning techniques [158] could further enhance adaptability to new tasks with minimal fine-tuning, making TLMs more versatile for broader applications. Another solution is the use of multi-task learning [159], where TLMs are trained simultaneously on multiple related tasks, encouraging generalization across contexts. Furthermore, few-shot and zero-shot learning techniques [160] could be employed to enhance the model’s performance in new domains without requiring extensive retraining. Incorporating dynamic, task-specific embeddings that adjust based on input context [161] can also help TLMs retain flexibility and adaptability across various applications.

### 5.3. Data Efficiency and Training Constraints

TLMs often require carefully curated training datasets and advanced training techniques such as knowledge distillation, quantization, or pruning to achieve high performance, which can be resource-intensive, particularly for smaller organizations. To alleviate data and resource constraints, data-efficient training strategies like semi-supervised learning [162] and synthetic data generation [163] can augment training data cost-effectively. Additionally, collaborative training [164] and open-source data-sharing platforms [165] can support teams in acquiring diverse datasets for training TLMs. Other approaches include federated learning [166], which allows models to be trained across decentralized datasets without sharing data, and transfer learning [167], where pre-trained models are fine-tuned on smaller, domain-specific datasets to save resources. Leveraging automated data augmentation techniques, such as back-translation or paraphrasing [168], can also help improve data diversity without incurring significant costs.

### 5.4. Ethical and Security Concerns

Deploying TLMs on edge devices and mobile platforms introduces privacy and security concerns, as models may process sensitive data directly on the device, raising issues around data responsibility and vulnerability to adversarial attacks. Ensuring secure deployment involves implementing on-device encryption and robust access control measures [169], while adversarial training [170] and model regularization [171] can bolster resistance to manipulation, enhancing security and privacy in TLMs deployment. Techniques such as differential privacy [172] can be integrated into the models to prevent information leakage, while secure multi-party computation [173] allows for privacy-preserving computations on shared data. Additionally, employing frequent security audits and monitoring systems for anomaly detection [174] can help identify and mitigate potential vulnerabilities early, providing robust protection against various types of adversarial attacks.

### 5.5. Reliability in Real-Time Applications

Although TLMs are designed for real-time applications, their reduced size can occasionally lead to inconsistent response quality, especially in complex interactions, affecting reliability in critical contexts like healthcare diagnostics or industrial automation. To improve reliability, ensemble methods combining multiple TLMs or hybrid models can be employed, with additional real-time error-checking and response fallback mechanisms [175] that provide more consistent outputs in applications where accuracy and consistency are critical. Implementing adaptive confidence thresholds can help the model defer to larger models or human operators in uncertain scenarios. Leveraging real-time monitoring tools to assess model output quality dynamically can also provide corrective feedback mechanisms that adjust responses based on context. Furthermore, regular retraining with up-to-date data and implementing redundancy within mission-critical systems [176] can improve reliability and consistency over time.

These challenges highlight the current limitations of Tiny Language Models and the ongoing need for research into methods that can enhance their performance, reliability, and ethical deployment across diverse application domains.

## 6. Future Directions for TLMs in Automation

As Tiny Language Models continue to evolve, there are several promising areas of research and development that could significantly enhance their effectiveness in automation and control. This section explores three primary directions: hybrid and adaptive compression techniques, application-specific TLMs, and context-aware, hardware-specific models. Each direction addresses specific challenges in deploying TLMs in real-world settings, with a focus on improving performance, efficiency, and applicability in diverse industrial environments.

### 6.1. Hybrid and Adaptive Compression Techniques

With the growing demand for ultra-compact and efficient models, researchers are exploring hybrid and adaptive compression techniques that combine multiple methods such as quantization, pruning, and knowledge distillation to create highly efficient TLMs without sacrificing accuracy.

*Dynamic Model Scaling for IoT Devices:* Hybrid compression techniques can allow TLMs to adjust their computational complexity based on the available resources of IoT devices. For instance, an industrial sensor could dynamically adjust its TLMs model size based on the remaining battery life, reducing power consumption when resources are limited.

*Adaptive Model Compression in Autonomous Vehicles:* Autonomous vehicles require both high efficiency and real-time processing for safe operation [177]. By leveraging hybrid compression, TLMs can prioritize specific aspects of processing, such as object detection and decision-making [178], to meet the immediate demands of different driving environments, balancing power and performance as needed.

*Low-Latency Processing in Edge Devices:* In applications where real-time responsiveness is critical, such as in automated quality inspection in manufacturing [179], adaptive compression enables TLMs to selectively reduce precision or apply sparsification based on current processing loads, ensuring rapid response without significant accuracy loss.

### 6.2. Application-Specific TLMs

To meet the specialized needs of distinct fields, future TLMs could be tailored for specific applications, such as industrial control, robotics, and healthcare. These application-specific TLMs would be optimized with domain-specific knowledge and fine-tuned for particular tasks.

*TLMs for Industrial Control Systems:* In manufacturing, TLMs customized for industrial control can assist in monitoring and optimizing production processes. For instance, TLMs fine-tuned for quality assurance could analyze visual data from cameras on the production line to detect defects in real-time, flagging irregularities with high precision.

*Robotics and Autonomous Operations:* Robotics applications, such as warehouse automation and robotic surgery [180], require precise language understanding and action guidance. TLMs designed specifically for robotics could interpret natural language instructions and convert them into safe, executable actions, such as guiding an autonomous robot to ‘pick up item X’ or ‘navigate to location Y’ efficiently within a constrained space.

*Healthcare Diagnostics:* In healthcare, TLMs fine-tuned on medical data can assist in diagnostics by interpreting clinical notes or patient queries. For example, TLMs tailored for radiology could analyze physician notes alongside diagnostic imaging data, aiding in the detection of anomalies and providing clinical insights for faster decision-making.

### 6.3. Context-Aware and Hardware-Specific Models

As TLMs become more integrated into various automation systems, there is a growing need for context-aware and hardware-specific models. These models would be designed to optimize performance for the environments in which they operate, considering both the physical context and the capabilities of the underlying hardware.

*Environment-Sensitive TLMs for Smart Cities:* In smart city applications [181], TLMs that understand the context such as traffic density or weather conditions could optimize city infrastructure management. For example, context-aware TLMs could adjust traffic signal timings during peak hours based on real-time traffic data, improving urban mobility and reducing congestion [182].

*Energy-Efficient TLMs for Battery-Powered Devices:* For devices like portable medical instruments or wearable sensors [183], TLMs that are specifically optimized for low-power hardware are essential. These models can reduce computation frequency or switch to low-energy processing modes, extending battery life while maintaining essential functions, such as continuous patient monitoring.

*Hardware-Optimized TLMs for Industrial Automation:* Industrial environments often employ custom hardware configurations, such as FPGAs and specialized processors [184]. Hardware-specific TLMs designed to leverage these platforms can accelerate processing times for tasks like predictive maintenance, enabling immediate anomaly detection and response on the factory floor.

The future of TLMs in automation will likely be shaped by advances in compression techniques, application-specific tuning, and context/hardware adaptability. These developments will expand the scope of TLMs applications, making it possible to deploy efficient, responsive language models across diverse fields and under various operational constraints, ultimately transforming automation and control systems.

## 7. Conclusions and Future Directions

The rapid evolution of NLP has been largely driven by the development of LLMs. While these models demonstrate remarkable capabilities across various applications, their substantial computational demands and memory footprints hinder their deployment on resource-constrained devices. To bridge this gap, TLMs have emerged as a compelling alternative, offering a balance between efficiency and performance. This survey has provided a detailed exploration of TLM architectures, training methodologies, optimization techniques, and their potential applications in automation and control.

TLMs leverage model compression techniques such as knowledge distillation, quantization, and pruning to retain essential language understanding capabilities while significantly reducing computational overhead. Their adoption across *edge computing*, *industrial automation*, *IoT*, and *healthcare* highlights their potential to facilitate real-time, low-power NLP applications. Furthermore, specialized *domain-specific TLMs*, such as those designed for scientific, legal, or medical contexts, are paving the way for more targeted, efficient language models that maintain domain relevance while ensuring computational feasibility.

Despite their promise, TLMs face notable challenges, including trade-offs between *model size and accuracy*, *generalization limitations*, and *ethical considerations* related to bias and privacy. Addressing these challenges necessitates further research into *hybrid compression strategies*, *knowledge fusion techniques*, and *energy-efficient model training*. Additionally, advancements in *hardware-aware model design*, *neuromorphic computing*, and *federated learning* could significantly enhance the deployment efficiency of TLMs in distributed and privacy-sensitive environments.

Looking ahead, the future of TLMs lies in their ability to *adapt dynamically to diverse applications*, integrate *context-aware optimizations*, and support *multimodal processing* beyond text-based tasks. The continuous refinement of *lightweight architectures*, *efficient training paradigms*, and *specialized datasets* will further propel TLMs toward *wider adoption in real-world scenarios*, reinforcing their role as a crucial component of next-generation NLP systems.

In summary, while *LLMs continue to push the boundaries of artificial intelligence*, TLMs offer a scalable, efficient, and sustainable alternative tailored for *real-time, on-device, and edge-based applications*. Future innovations in *algorithmic efficiency*, *hardware co-optimization*, and *transfer learning* will further strengthen their utility, making them a key driver in the democratization of AI-powered language understanding and automation.

## Figures and Tables

**Table 1:** Comparison of model size reduction techniques with defined metrics.

Technique	Description	Model Size Reduction	Latency Improvement	Accuracy Trade-Off
Knowledge Distillation	Teacher-student model transfer	High (>50%)	Moderate (1.2×–2×)	Minimal (<1%)
Quantization	Lower precision (e.g., INT8)	High (>50%)	High (>2×)	Minor (1–3%) for INT8
Pruning	Remove unimportant weights	Moderate (20–50%)	Moderate (1.2×–2×)	Variable
Efficient Architectures	Model design for efficiency	High (>50%)	High (>2×)	Minimal (<1%) if optimized

**Table 2:** Comparison between LLMs and TLMs (based on quantitative and qualitative analysis).

Aspect	LLMs	TLMs
**Model Size**	Billions of parameters (e.g., GPT-3: 175 B; PaLM: 540 B)	Tens to hundreds of millions (e.g., DistilBERT: 66 M; TinyBERT: 4.4 M)
**Computational Requirements**	Extremely high: Requires multiple GPUs or TPUs, often in cloud environments	Moderate to low: Operates efficiently on single GPUs or CPUs
**Latency**	High (e.g., GPT-3 inference latency 100–200 ms for cloud-based tasks)	Low (e.g., optimized for <50 ms response times in edge environments)
**Energy Efficiency**	High energy consumption (e.g., training GPT-3 requires hundreds of MWh)	Optimized for low-power settings (e.g., inference on mobile devices)
**Memory Footprint**	Very large (requires distributed storage or advanced caching techniques)	Compact (fits within edge device memory, typically < 1 GB)
**Accuracy and Performance**	High on complex tasks (e.g., SOTA on NLP benchmarks)	Moderate to high; some accuracy trade-off for efficiency
**Training Techniques**	Pre-training on massive datasets, extensive fine-tuning	Model compression (e.g., distillation, pruning, quantization)
**Deployment Scope**	Cloud-based or server environments	On-device, edge, IoT, or mobile platforms
**Inference Speed**	Variable; often slow for large-scale models due to size	Fast; optimized for low-latency environments
**Suitability for Real-Time Applications**	Limited due to latency and resource constraints	Ideal for real-time applications, especially on edge devices
**Common Use Cases**	Large-scale NLP (e.g., machine translation, chatbots, summarization)	Resource-efficient NLP (e.g., voice assistants, mobile diagnostics)
**Relevance in Automation**	High in centralized processing pipelines	High for real-time, localized automation
**Hardware Compatibility**	Requires specialized hardware (e.g., TPU, GPU clusters)	Compatible with standard hardware (e.g., CPUs, low-power GPUs)
**Cost of Deployment**	Very high: Significant investment in infrastructure and cloud usage	Low: Affordable for small-scale or local deployments

**Table 4:** Key applications of TLMs in automation and control.

Application Domain	Function of TLMs	Expected Benefits
Smart Home Assistants	On-device NLP for automation	Low-latency, privacy-friendly voice interaction
Wearable Health Monitors	Real-time health insights processing	Improved accessibility and energy efficiency
Industrial IoT Monitoring	Sensor data analysis and anomaly detection	Enhanced predictive maintenance and uptime
Smart Agriculture Systems	AI-powered environmental monitoring	Increased resource efficiency and automation
Voice-Controlled Manufacturing	Hands-free machine operation	Simplified workflow and human-machine interaction
Automated Industrial Reporting	NLP-based log analysis and summarization	Reduced manual reporting efforts and faster insights
Multilingual NLP for Factories	Real-time translation of operator commands	Enhanced collaboration in global workplaces
Error Code Interpretation	AI-based fault diagnosis	Faster troubleshooting and maintenance resolution

## Data Availability

No new data were created or analyzed in this study. Data sharing is not applicable to this article.
